# Influence of Solid Retention Time on Membrane Fouling and Biogas Recovery in Anerobic Membrane Bioreactor Treating Sugarcane Industry Wastewater in Sahelian Climate

**DOI:** 10.3390/membranes13080710

**Published:** 2023-07-31

**Authors:** Abdoul Wahab Nouhou Moussa, Boukary Sawadogo, Yacouba Konate, Brony Thianhoun, Sayon dit Sadio Sidibe, Marc Heran

**Affiliations:** 1 Laboratoire Eaux Hydro-Systèmes et Agriculture (LEHSA), Institut International d’Ingénierie de l’Eau et de l’Environnement (2iE), Rue de la science, Ouagadougou 01 BP 594, Burkina Faso; boukary.sawadogo@2ie-edu.org (B.S.); yacouba.konate@2ie-edu.org (Y.K.); brony.thianhoun@2ie-edu.org (B.T.); 2 Laboratoire Energies Renouvelable et Efficacité Energétique (LaBEREE), Institut International d’Ingénierie de l’Eau et de l’Environnement (2iE), Rue de la science, Ouagadougou 01 BP 194, Burkina Faso; sayon.sidibe@2ie-edu.org; 3 Institut Européen des Membranes (IEM), UMR-5635, Université de Montpellier, CNRS, Place Eugène Bataillon, CEDEX 5, 34095 Montpellier, France; marc.heran@umontpellier.fr

**Keywords:** acclimatization, industrial wastewater treatment, methane production, operating conditions, transmembrane pressure

## Abstract

Sugarcane industries produce wastewater loaded with various pollutants. For reuse of treated wastewater and valorization of biogas in a Sahelian climatic context, the performance of an anaerobic membrane bioreactor was studied for two solid retention times (40 days and infinity). The pilot was fed with real wastewater from a sugarcane operation with an organic load ranging from 15 to 22 gCOD/L/d for 353 days. The temperature in the reactor was maintained at 35 °C. Acclimatization was the first stage during which suspended solids (SS) and volatile suspended solids (VSS) evolved from 9 to 13 g/L and from 5 to 10 g/L respectively, with a VSS/SS ratio of about 80%. While operating the pilot at a solid retention time (SRT) of 40 days, the chemical oxygen demand (COD) removal efficiency reached 85%, and the (VSS)/(TSS) ratio was 94% in the reactor. At infinity solid retention time, these values were 96% and 80%, respectively. The 40-day solid retention time resulted in a change in transmembrane pressure (TMP) from 0.0812 to 2.18 bar, with a maximum methane production of 0.21 L/gCOD removed. These values are lower than those observed at an infinite solid retention time, at which the maximum methane production of 0.29 L/gCOD was achieved, with a corresponding transmembrane pressure variation of up to 3.1 bar. At a shorter solid retention time, the fouling seemed to decrease with biogas production. However, we note interesting retention rates of over 95% for turbidity.

## 1. Introduction

In the context of human nutrition, sugar holds a prominent position, as evidenced by the average consumption of approximately 25 kg per annum per person. Its production primarily involves the use of sugar beet or sugar cane as raw materials in various industries [1,2,3,4,5]. Accounting for more than 70% of global sugar production, sugar cane is the most significant source of sugar worldwide. This crop is cultivated on more than 22 million hectares of land in over 100 countries [6]. The various sugar processing operations generate large quantities of wastewater, which, in addition to water recovery, can be a source of nutrients or energy. To meet this challenge, several treatment systems are used to treat such wastewater. Physicochemical treatment systems offer pollution removal rates above 90%, but their limitations lie in the production of secondary compounds due to the use of chemicals [7]. Other treatment methods utilizing microorganisms with a biological process can also eliminate organic pollution, albeit with limited efficacy in removing heavy metals and recalcitrant compounds [8,9]. Balancing the different treatment systems is crucial, given the high pollutant loads present in sugarcane industry effluent. The use of membrane bioreactors stands out as a promising treatment method for sugarcane industry effluents, with numerous benefits [10,11].

A membrane bioreactor integrates biological treatment with a reactor that eliminates biodegradable pollution and retains suspended solids and certain inorganic compounds using a porous membrane. Moreover, a membrane bioreactor provides several benefits, including a small operational footprint, low sludge generation, and excellent removal rates of organic pollutants, also allowing for a long solid retention time (SRT) due to the decoupling of hydraulic retention time. Furthermore such bioreactors permit the treatment of nitrogen and other more recalcitrant compounds [12,13]. Membrane bioreactors provides the additional advantage of biogas recovery during anaerobic operation [14,15]. Biogas yield is influenced by temperature and solid retention time (SRT), both of which are strongly tied to substrate conversion in the reactor.

At low temperatures, microbial population growth, biological reactions, and biogas production all suffer [16,17]. Thermophilic temperatures promote hydrolytic activity and the elimination of recalcitrant compounds; however, they also lead to a substantial decrease in the microbial population diversity responsible for pollutant breakdown. Consequently, mesophilic reactors that can withstand operational disturbances are favored [18]. An optimum temperature of approximately 35 °C is recommended for anaerobic treatment with biogas recovery [19,20]. The Sahelian climatic context would therefore be suitable for anaerobic digestion of sugarcane industry effluents, with temperatures above 30 °C [21,22].

One of the major challenges in wastewater treatment with a membrane bioreactor is fouling, which can reduce the effectiveness of the membrane filtration process and increase operational costs [23,24,25]. Membrane fouling is the unwanted buildup and accumulation of microorganisms, colloids, solutes, and cell debris on or within membranes [26,27]. This phenomenon is more complex in membrane bioreactors (MBRs) than in most other membrane applications due to the intricate nature of activated sludge [28]. Membrane bioreactors are subject to various membrane fouling mechanisms, such as the adsorption of colloids or solutes on or within the membranes, the accumulation of sludge particles on the surface of the membrane, and the development or detachment of a cake layer on the surface of the membrane [29]. Membrane cleaning typically entails physical, chemical, and biological methods, which are selected based on the membrane materials, foulant composition, and cleaning reagents. Although physical cleaning is more frequently employed, its efficacy may be limited in cases of irreversible fouling. It involves in-line ultrasonic cleaning, carrier cleaning, purging, backwashing, aeration, and swinging [30]. During chemical cleaning, solutions of acids or bases are used, whereas biological enzymes and bacterial QQ are sufficient for removing fouling during biological cleaning [31,32]. The permeate or recovery biogas can be recycled for membrane backwashing. Activated carbon or other absorbent materials can be added to the reactor to combat fouling [33,34]. Backwashing assists in reducing membrane fouling and restoring filtration efficiency, while membrane cleaning is a more intensive process conducted periodically or as necessary to eliminate stubborn or persistent fouling from the membrane surface. Higher flux rates can elevate the risk of fouling as a result of increased contact between the membrane and contaminants. Similarly, although high TMP can enhance filtration performance, it may also contribute to fouling if not adequately controlled [19,35,36]. The energy required for either backwashing or cleaning raises both operating and maintenance expenses [13]. A multiscale approach would allow for a better understanding of the complex phenomenon whereby proteins embed into membranes during filtration. These models can also be used to predict the adsorption energy or the orientation of the enzyme on the membrane [37]. By transitioning from a microscopic scale to a macroscopic scale and formulating a non-stationary mass transfer model, the behavior of the ultrafiltration process can be described [38]. Asiri et al. [39] argued that fouling can be reduced by modifying a cellulose acetate membrane with ZnO. Replacing the membrane with a cellulose acetate membrane can make membrane bioreactors strong contenders for removing color from wastewater [40].

The solid retention time (SRT) is another operational parameter that has a direct impact on both fouling and biogas production, as well as the overall performance of membrane bioreactors. However, few studies have investigated the influence of SRT on the treatment of sugarcane industry effluents using anaerobic membrane bioreactors [41,42]. The choice of SRT must be judicious, as shortening or prolonging it could directly influence the performance of the treatment. Dong et al. [43] observed reductions in SS, COD concentration, and fouling in a bioreactor at an SRT < 40 days. A long SRT increases fouling by promoting biogas production and the development of new bacterial populations in the reactor [44]. A balance must be found to compensate for fouling, biogas production, and treatment performance when selecting the SRT for wastewater treatment with a membrane bioreactor.

By varying the SRT at a temperature of 35 °C, in the present study, we analyzed the effect of the SRT on the performance of a membrane bioreactor under anaerobic conditions. Therefore, this is an original study using real effluents from sugarcane industries, taking into account the effects of effluents from both a sugar mill and a distillery on a technology whose applications in the Sahelian context are rather rare, while the local ambient temperature should only encourage them.

## 2. Material and Methods

In this study, an anaerobic membrane bioreactor with a ceramic membrane was operated continuously without interruption under three stages of variations in operating conditions. Biogas production was also evaluated during the treatment. Various performance parameters were monitored using physical, biological, and chemical analyses.

### 2.1. Experimental Setup

The study was carried out using an Altuglas anaerobic membrane bioreactor. The total reactor volume was 23 L, operating with a useful volume of 20 L (87% of the total volume). The pilot was fed by pulse at a regular rate with a peristaltic pump from an effluent storage tank. The feeding solution was real wastewater from a sugar and alcohol industrial plant located in the southwestern region of Burkina Faso.

**Figure 1:**
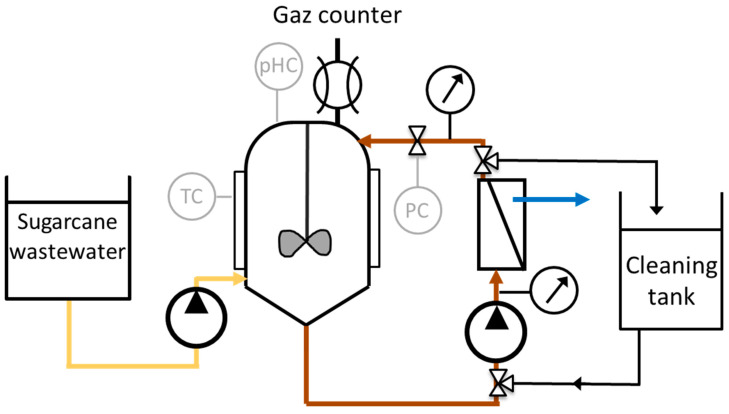
Schematic diagram of the reactor.

The membrane was installed in a cartridge outside the bioreactor with a tangential filtration system (in–out). The retentate was recirculated into the reactor by a volumetric pump, allowing for adjustment of the tangential velocity along the membrane. The membrane was a P10 ceramic membrane module provided by Pall Exekia, with a filtering surface of 0.45 m^2^. The cut-off threshold was between 10 and 20 nm at 15 kD. The total length of the membrane was 1178 cm, with 20 channels of 6 mm diameter. pH, temperature, and transmembrane pressure were monitored and controlled using a software system, as shown in Figure 1. The software enabled the display of various parameters, such as pressure, flow, pH, and temperature, providing a convenient interface for monitoring of the membrane bioreactor. The pH was regulated by the addition of NaOH (sodium hydroxide). The experiments were conducted under Sahelian climatic conditions, with temperatures exceeding 30 °C [45]. Consequently, the temperature inside the reactor was maintained at 35 °C through the implementation of a cooling unit. Additionally, the transmembrane pressure (TMP) was regulated by employing a back pressure valve positioned downstream of the membrane.

As far as clogging is concerned, a periodic wash was carried out depending on the transmembrane pressure.

The cleaning process involved using chemical solutions to wash the membrane. Specifically, a solution consisting of 2% NaOH, 400 ppm active chloride, and 1 N nitric acid was prepared. The cleaning solutions were introduced into a designated cleaning tank, then fed into the membrane. Subsequently, the membrane was rinsed with prefiltered water to ensure thorough cleaning. A schematic representation of this cleaning process can be found in Figure 1.

The first step involved cleaning with NaOH solution for 30 min, followed by a 15 min rinse with nitric acid. The membrane was then washed with activated chloride for a period of 30 min before being rinsed with prefiltered water.

### 2.2. Sampling and Analytical Methods

To assess the performance of the AnBRM, several parameters were monitored in the reactor and the permeate. Monitoring of the membrane also included tracking variations in pressure and flow surrounding the membrane. pH variations were measured throughout the operation, along with the flow rate. This comprehensive monitoring approach ensured that changes in pressure, flow, and pH were closely monitored and recorded during the membrane operation. Samples were routinely collected from the factory, the bioreactor, and the permeate three times per week for analyses according to standard methods for examination of water and wastewater [45,46,47], as described in Table 1.

A continuous gas meter (Milligas counter RITTER) was used to monitor biogas production, which was collected in Tedlar bags for analysis by an OPTIMA7 Analyzer to determine the biogas composition. The influent was neutralized with slaked lime from the sugarcane industry, and its characteristics are presented in Table 2.

### 2.3. Operating Conditions

The sludge used as inoculum for the anaerobic membrane bioreactor was sourced from a biogas plant located in the industrial zone of Ouagadougou. This particular plant employs biodigesters to convert slaughterhouse waste and other organic substrates into electricity and biofertilizers. The choice of this sludge was not arbitrary but based on the presence of methanogenic bacteria, which are crucial for efficient anaerobic digestion. Utilizing this sludge as the inoculum offered significant advantages due to its effective population of methanogenic bacteria, making it a favorable choice for the bioreactor system. For startup, the bioreactor was inoculated with 20% anaerobic sludge and supplemented with the feed substrate [21,48,49].

During the 353-day operation of the pilot, several parameters and stages were monitored. The hydraulic retention time (HRT) remained constant at 40 h throughout the entire operation, maintaining a temperature of 35 °C. The SRT was set at 40 days for one stage, then at infinity for another stage to evaluate and compare the performance of the membrane bioreactor. The tests were conducted in three stages. Initially, there was a 62-day acclimation period, after which the pilot operated with a 40-day SRT for 146 days. Subsequently, the SRT was extended to infinity for another 146 days. By varying the SRT, the impact on the system’s performance could be evaluated. Throughout the 353-day operation, the filtration flux remained constant at 1.11 LMH in order to assess the variations in transmembrane pressure and membrane fouling over time. The organic load in the feed solution ranged from 15 to 22 gCOD/L/d, with COD concentrations ranging from 26 g/L to 37 g/L. This comprehensive operational period allowed for a thorough evaluation of the membrane bioreactor’s performance under different SRT conditions and organic load variations, providing valuable insights into transmembrane pressure and membrane fouling.

Regarding the mass balance, the sludge production was calculated according to Equation (1). The first term describes the sludge production, which is output with the sludge extraction, whereas the second term describes the sludge accumulation in the biological tank. This second term was calculated according to the evolution of VSS between each time slot (Δt) or simply by measuring the slope of VSS(t).

(1) $$P_{Sludge}=Q_{w}VSS_{Reactor}+V_{Reactor}\frac{\Delta VSS_{Reactor}}{\Delta t}\;$$

The key performance indicators of the membrane bioreactor during the operation are as follows:

$$\frac{\mathrm{VSS}}{\mathrm{SS}}\left(\%\right)=\frac{\mathrm{VSS}}{\mathrm{SS}}*100$$

$$\mathrm{COD}\;\mathrm{removal}\;\mathrm{rate}\;\left(\%\right)=\frac{\mathrm{CODin}-\mathrm{CODeff}}{\mathrm{CODin}}*100$$

$$\mathrm{Ions}\;\mathrm{removal}\;\mathrm{rate}\;\left(\%\right)=\frac{\left[\mathrm{Ions}\right]\mathrm{in}-\left[\mathrm{Ions}\right]\mathrm{eff}}{\left[\mathrm{Ions}\right]\mathrm{in}}*100$$

$$\mathrm{Methane}\;\mathrm{specific}\;\mathrm{yield}\;\left(\frac{\mathrm{LCH}4}{\mathrm{gCOD}}\right)=\frac{\mathrm{Volume}\;\mathrm{CH}4}{\mathrm{ORL}}$$

where:

VSS: concentration of volatile suspended solids;
SS: concentration of suspended solids;
CODin: COD influent;
CODeff: COD effluent;
$\left[\mathrm{Ions}\right]\mathrm{in}$ : ion concentration in the influent;
$\left[\mathrm{Ions}\right]\mathrm{eff}$ : ion concentration in the effluent;
Volume CH_4_: methane volume per day;
OLR: organic loading rate.

## 3. Results and Discussion

### 3.1. Biomass Acclimatization and Evolution

The SS and VSS concentrations in the inoculum were 6.33 and 5.16 g/L, respectively. The effluent values ranged from 7.32 to 8.96 g/L for SS and from 5.01 to 6.12 g/L for VSS.

**Figure 2:**
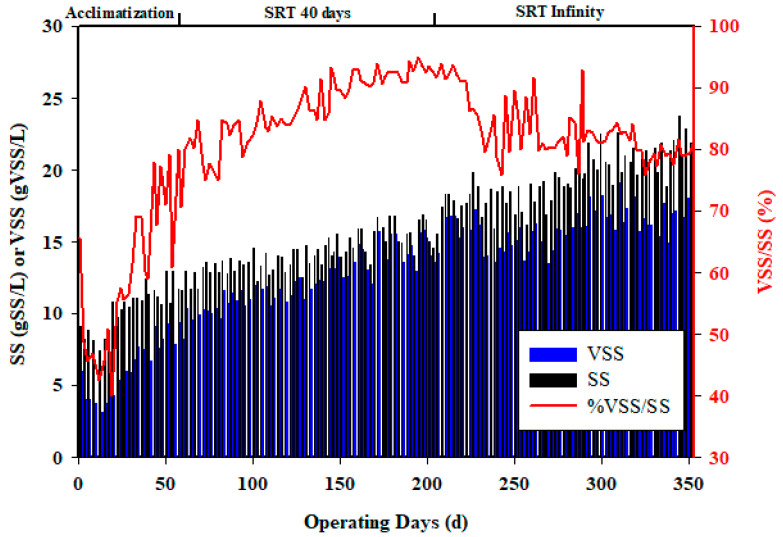
Evolution of SS and VSS concentration during experimental periods.

Figure 2 depicts the progression of SS and VSS throughout the operation. These findings align with the results of a study conducted by Casu et al. [48], who observed an increase in SS levels from 5.5 g/L to 20.4 g/L without sludge removal when treating wastewater using a submerged anaerobic membrane bioreactor. During the initial period (day 1 to day 62), when no sludge extraction was performed, the SS and VSS concentrations within the reactor experienced continuous and substantial growth. Specifically, the SS concentration rose from 7.4 g/L to 23.8 g/L, while the VSS concentration increased from 4.7 g/L to 19.5 g/L. These findings highlight the significant and continuous accumulation of suspended solids within the system in the absence of sludge removal. The acclimatization period consisted of a gradual buildup to VSS values of 10 g/L and a VSS/SS ratio of 80% [50,51]. The high levels of SS and VSS present in the influent, combined with the dilution of the inoculum (which accounted for 20% of the total volume), resulted in a slower acclimatization process compared to the findings reported in a study conducted by Sawadogo et al. [21], in which acclimatization was applied for approximately 20 days by treating wastewater from beverage production operations with an initial sludge load of 6 g/L SS.

The presence of foam was observed at the beginning of the pilot, indicating that the stress experienced by the bacteria was responsible for organic pollution degradation. During this initial period, a decline in VSS concentration was observed before it started to increase again. The appearance of foam is often associated with process instability or an imbalance in the microbial community. In this case, the presence of suggested that the bacteria initially faced challenges in adapting to the conditions and effectively degrading the organic pollutants. Consequently, the VSS concentration initially decreased as the bacteria struggled to cope with the stress. However, as the system stabilized and the bacteria acclimated, the VSS concentration began to recover and increase, indicating a restoration of microbial activity and performance [26,29,52].

Other tests were carried out in order to better appreciate the acclimatization and the density of the microbial population [53,54,55].

From day 62 to day 208 of the operation, the pilot was operated with a solid retention time (SRT) of 40 days. During this period, both SS and VSS exhibited a regular evolution. Specifically, the SS concentration increased from 12.3 g/L to 16.5 g/L, while the VSS concentration increased from 10.4 g/L to 15.6 g/L. Interestingly, these values were higher in the reactor compared to the period with an infinity SRT (days 206 to 353). In the reactor with an infinite SRT, the SS concentration increased from 16.3 g/L to 23.2 g/L, and the VSS concentration increased from 15.6 g/L to 19.2 g/L. This slower change in VSS concentration in the reactor during the period with an infinity SRT is attributed to a significantly higher conversion rate of COD to biogas. This suggests that a longer SRT allows for more efficient conversion of organic matter into biogas, resulting in a lower accumulation of VSS in the reactor [56].

During the period of infinity SRT, there was no sludge extraction, resulting in the accumulation of inert inorganic substances in the reactor. This phase was marked by a decrease in the VSS to SS ratio, from 95% to 81%. This decrease can be attributed to two phenomena. First, there was a decrease in organic matter, as it was more efficiently converted into biogas, implying that a larger portion of the organic matter was transformed into biogas, leading to a decrease in its concentration in the form of volatile suspended solids. Secondly, there was an accumulation of mineral matter or inert inorganic substances in the reactor. These substances do not contribute to the VSS fraction, thus reducing the VSS/SS ratio. The accumulation of mineral matter occurs as a result of the absence of sludge extraction, allowing for the gradual buildup of inorganic substances over time. Together, these two phenomena, i.e., the decrease in organic matter transformed into biogas and the accumulation of mineral matter, contributed to the observed decrease in the VSS/SS ratio during the phase of infinity SRT.

### 3.2. Treatment Efficiency

**Figure 3:**
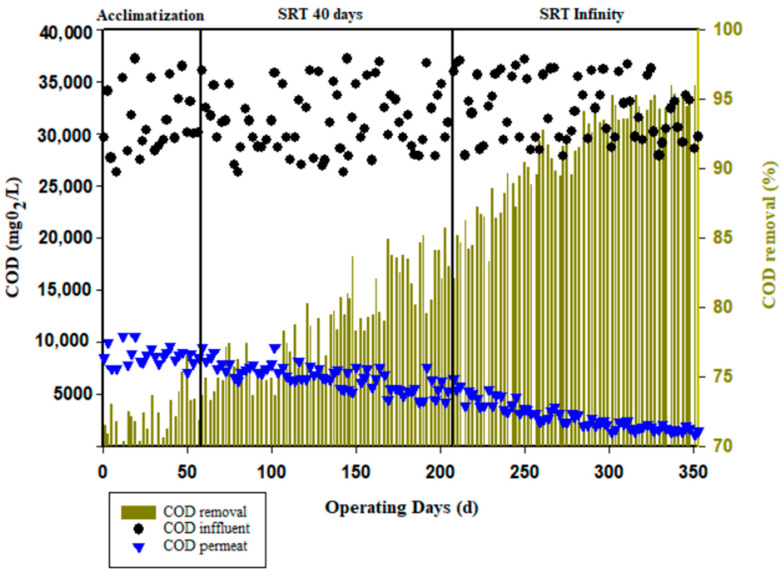
COD elimination in the AnBRM.

The AnBRM performance is reflected, on the one hand, by the VSS evolution in the reactor and, on the other hand, by the biogas production and the COD permeate concentration. The monitoring of COD in the reactor allowed us to assess the capacity of the reactor to degrade organic pollution during the operation. The COD concentration in the feed was between 26,230 and 37,170 mg/L. Throughout the operation, the membrane provided COD retention ranging from 61 to 75%. The total COD removal in the membrane bioreactor combined the biological activities in the reactor and the retention on the ultrafiltration membrane. Figure 3 illustrates the total COD removal AnBRM rate, ranging from 69% at the beginning of the experiment to 95% at the end. Vaquerizo et al. [57] achieved a COD removal of approximately 75% by treating vinasse in a membrane bioreactor at an organic load of 5.1 gCOD/L/d. Higher removal rates were obtained by Santos et al. [58] when treating vinasse with AnBRM: about 97% at an organic load of 6 gCOD/L/d and an SRT of 280 days. Several authors have reported COD removal rates above 95% using different bioreactor configurations and several effluents [59,60,61,62]. During the acclimatization period, the slow evolution of the removal rate is attributed to low biological activity. In fact, the reactor was only seeded with 20 % inoculum. The period just after acclimatization from the day 62 to day 206 of operation corresponding to the operation of the pilot at an SRT of 40 days was characterized by a continuous decrease in the permeate concentration. In the membrane bioreactor, the COD removal rate ranged from 73% to 85%. However, from day 206 to day 353, corresponding to an infinity SRT, the COD removal rate increased from 87% to 96%.

Altering the SRT does not have a significant impact on the removal of organic pollution. However, increasing the SRT allows microorganisms to degrade even the fraction that is challenging to biodegrade [63,64,65]. This point can be confirmed by assessing the production of biogas. In the same way, He et al. [66] reported a COD removal rate of 99.5% for a long SRT and 99% for a short SRT in wastewater treatment with 5 g/L phenol using a ceramic membrane bioreactor. In contrast, Nilusha et al. [67] showed that the higher the SRT, the lower the COD removal. Other researcher showed that the quality of the permeate is reduced by prolonging the SRT [68], which was not observed in the present study.

### 3.3. Water Quality

In distillery effluent, turbidity levels are typically very high [18,69,70]. The influent turbidity ranges from a minimum of approximately 1456 NTU to a maximum of 1966 NTU. During the acclimatization period, the turbidity reduction was quite low. The total turbidity removal by the membrane bioreactor was about 94 to 99%. The operation of the bioreactor with an infinity SRT had a negative impact on turbidity removal, primarily due to a lack of sludge drawdown. COD removal is not related to color removal, as most of the compounds responsible for vinasse coloring are recalcitrant compounds. Slightly higher turbidity removal rates of around 97% were achieved by Mota et al. [71]. Conductivity values in the feed solution varied between 10,020 and 12,960 µs/cm. The membrane bioreactor allowed us to achieve a total conductivity removal of 35 to 58%.

A significant elimination of sulfate ions (S0_4_^2−^) in the reactor from 66 to 91% was achieved. This degradation was caused by sulfidogenesis [72,73], which is nothing more than the reduction in the sulfate ion by hydrogen forming the hydrogen sulfide. The permeate of the membrane bioreactor exhibited sulfate ion (S0_4_^2−^) values ranging from 195 to 44 mg/L. Nitrate (NO_3_^−^) followed the same pathway, with nitrates reduced to nitrogen gas. The concentration of orthophosphate ions (PO_4_^3−^) in the effluent ranged from 70 to 98 mg/L. Santos et al. [58] achieved a removal efficiency of 79% for sulfate ions (SO_4_^2−^) and 30% for orthophosphate ions (PO_4_^3−^) using AnBRM applied to vinasse at a hydraulic retention time of 3.1 days.

**Figure 4:**
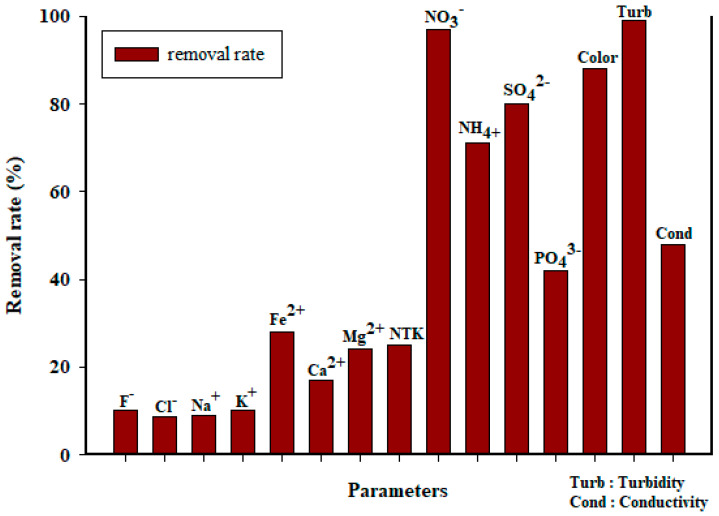
Removal of physicochemical parameters in the AnBRM.

The retention of monovalent ions such as chloride, fluoride, sodium, and potassium by the ultrafiltration membrane remained quite low throughout the treatment, not exceeding 10%. However, it should be noted that, particularly for the hydrogen carbonate ions (HCO_3_^−^), an accumulation in the reactor was observed, which can be explained by the production of hydrogen carbonate ions (HCO_3_^−^) in the reactor during the methanization. With initial values of 920 to 1085 mg/L and a retention on the membrane of around 12%, these values remained high in the permeate, up to 180% or 1764 mg/L. Divalent ions in the influent were more retained by the membrane compared to monovalent ions, with removal rates above 20%. The membrane bioreactor achieved a total removal rate of approximately 30% for certain ions, such as magnesium ions (Mg^2+^) and iron ions (Fe^2+^) as illustrated by Figure 4. Several authors have shown a limitation of membrane bioreactors in terms of ion removal [21,74].

### 3.4. Biogas Production

In an anaerobic membrane bioreactor offers the potential of recovering biogas. Theoretically, with 1 g of COD at 0° and a pressure of 1 atm, about 0.350 L of methane can be produced. It is possible to recover between 10 and 26.4 m^3^ of biogas with 1 m^3^ of vinasse with a methane composition of about 60%, as vinasse is essentially composed of biodegradable organic pollution [75]. The biogas produced in an anaerobic membrane bioreactor contains various other components or elements, mainly CO_2_ and H_2_S. The former reduces the calorific value of the biogas, while the latter is harmful to health and devices [76].

It should also be noted that water vapor in the biogas is corrosive to the pumps [77]. With high organic load at the inlet (15 to 22 g COD/L/d), the gross biogas production reaches 0.64 L of gross biogas per gram of COD removed. This corresponds to a specific methane production of 0.29 L_CH4_/g_COD_. During the acclimatization stage, the percentage of CH_4_ can increase from 65 to 76% in an acclimatized reactor and from 26 to 73% in a non-acclimatized reactor [45,78]. The acclimatization stage is characterized by average biogas production, with a methane percentage around 67%, which can be explained by an accumulation of TS in the reactor. The percentage of TS in the reactor was between 4 and 14%, with a maximum rate during the acclimation phase. The accumulation of TS in the reactor caused a subsequent buildup of VFA, as explained by Mota et al. [71].

When the concentration of VFA reaches 1000 mg/L, biogas production stops during the acidogenesis phase, leading to a decrease in pH.

During the period of TS accumulation, the VFAs in the reactor reached a concentration of 489 mg/L. Starting from day 58, a continuous decrease in TS was observed within the reactor, which is attributed to the increased removal rate of organic pollution. The increase in the COD removal rate is closely associated with the reduction in VFAs in the reactor, as TS accounts for a significant portion of organic pollution. Methanization evolves before the end of acclimatization, whereupon biogas production increases in proportion to the percentage of CH_4_. During the operation of the pilot with an infinity solid retention time (SRT), the biogas production in the reactor contained a high percentage of CH_4_ compared to the period when the SRT was 40 days. During the period from day 62 to day 206 of operation, a specific methane production of 0.21 L biogas/gCOD was observed. Subsequently, from day 206 to day 353 of operation, the specific methane production increased to 0.29 LgDCO/L/d. It appears that methane production increases with a long SRT. Lovato et al. [79] reported a CH_4_ percentage of 82.3% when treating cane vinasse with a membrane bioreactor. Chen et al. and Svojitka et al. [80,81] reported more than 0.2 LCH_4_/gCOD/d when treating industrial wastewater, while Turker et al. and Balcıoğlu et al. [82,83] reported CH_4_ volumes below 0.3 L of CH_4_/gCOD

In order to further investigate the biomass evolution and gas production, several mass balances were implemented to understanding the COD pathway: only retained by the membrane, transformed into biogas, and leaving the reactor in the sludge or permeate (Table 3).

**Figure 5:**
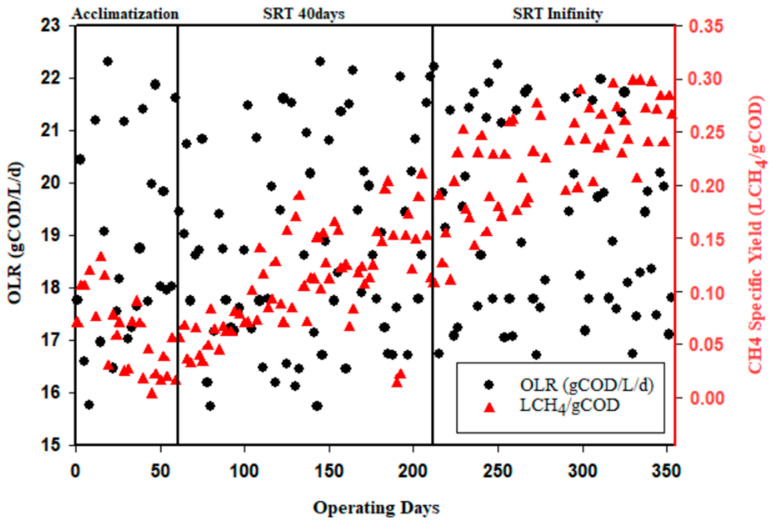
Organic load and CH_4_ production in the reactor.

As the operating time was extended to values approaching the theoretical ratio of 0.35 L CH_4_/gCOD, the production of biogas increased, which implies that all the retained COD was transformed into biogas. The COD was initially accumulated in the reactor in the form of VSS and subsequently converted into biogas during the operation. When this accumulation was limited, even without sludge extraction, the VSS increased only slightly (Figure 2). The obtained results are summarize in Table 4. The reactor is capable of handling a very high organic loading rate (>19 kg_COD_/m^3^/d) with a very high efficiency in terms of organic matter removal, confirming the relevance of AnMBR. The concentration of the outlet water was low (1500 mg_COD_/L), but the reactor does not seem to have reached a steady state, even after more than 350 days of operation, as the COD effluent still decreased over time (Figure 3). The conversion yield of gas production (Figure 5) per liter of methane per kg of COD removed was close to the theoretical value, which implies that the main mechanism is a biological transformation and not retention by the membrane. The organic sludge conversion yield in kg of VSS formed per kg of COD removed was very low, confirming the value of anaerobic processes to significantly reduce sludge production and the related cost for sludge disposal. The overall performances are presented in Table 5.

### 3.5. Biomass Investigation

**Figure 6:**
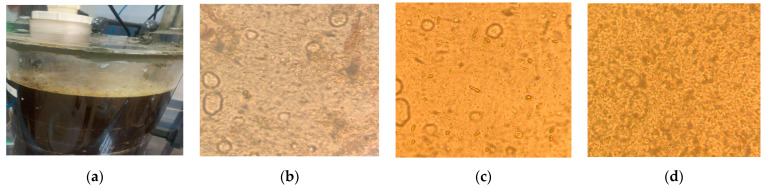
Observation of microorganism density with an epifluorescence microscope (×40 magnification). (**a**) Microbial stress in the reactor; (**b**) observation on day 1 (**c**); observation on day 30; (**d**) observation on day 62.

**Figure 7:**
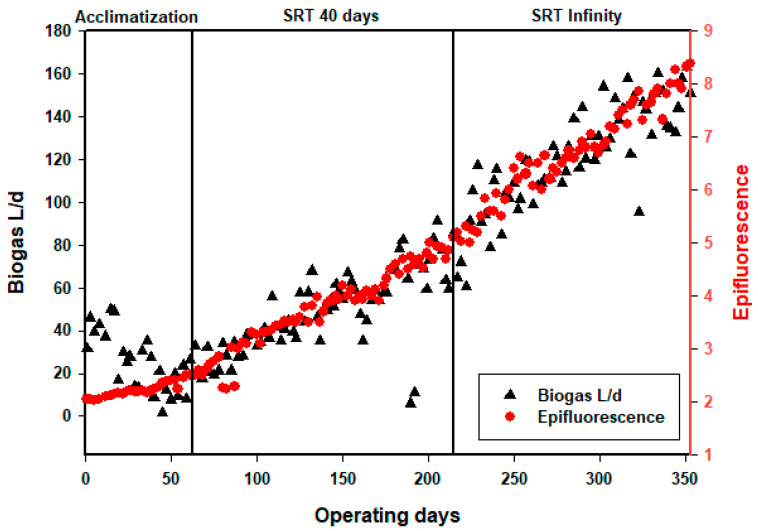
Measurement of absorbance and biogas production with a DR3900 spectrophotometer.

In order to confirm the acclimatization of microorganisms, the biomass conversion yield, and methane conversion yield, measurements were taken using an epifluorescence microscope at a wavelength of 600 nm on a molecular absorption spectrophotometer. Microscope observation helps to elucidate the behaviors of the microorganisms, while measurements with the DR3900 spectrophotometer translate the density of the biomass and its temporal evolution. The evolution of the biomass can therefore be observed with a grouping of microorganisms in the form of flocs to trap the organic pollution and facilitate its degradation. Microorganisms clustered in fine flocs affect the removal of organic pollution [84]. This evolution is also reflected in the size of the microorganisms and the size of the flocs. During operation, the microorganisms aggregate into larger flocs, and their population increases, leading to an overall growth in their number. This evolution was followed by three different periods: at startup, after one month of operation, and at the end of acclimatization. At startup, we observed microorganisms grouped in small flocs and often dispersed. After 30 days of operation, the microorganisms began to aggregate and form medium-sized flocs. At the end of the acclimatization period, after 62 days of operation, the microorganisms were grouped in denser flocs. The small flocs will led to a deterioration of the reactor performance [84]. These different stages of microorganism evolution are represented in Figure 6. As far as the observation with the spectrophotometer is concerned, an increasing curve with progressively higher absorbance values was observed, indicating a growing concentration or intensity of the measured parameter [55]. The measurements were carried out twice a week in order to appreciate the evolution of the microbial density. After each measurement, an increase in the bacterial population was observed, as reflected by an increase in the absorbance values. At the beginning of the operation, the absorbance was measured at 2.04, which gradually increased over time and reached 8.32 by the end of the operation. This evolution of absorbance is depicted in Figure 7. The utilization of these two methods provides a clear understanding of the biomass evolution.

### 3.6. Fouling and Membrane Filtration Performance

**Figure 8:**
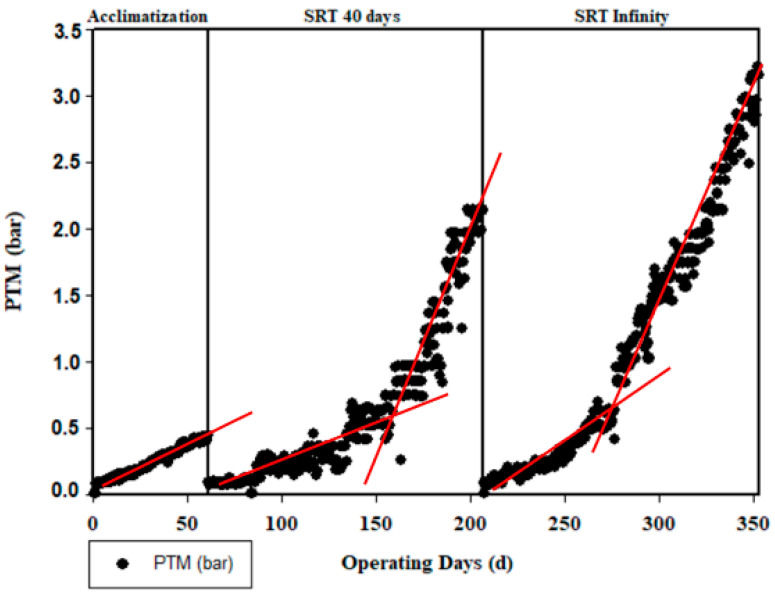
Transmembrane pressure evolution.

Due to the constant flow rate during the filtration operation, the TMP gradually increased over time, as shown in Figure 8. This increase in TMP is attributed to fouling, which involves the formation of a layer around the membrane. If the layer is formed by the accumulation of suspended solids, it is referred to as a cake. On the other hand, the term biofilm is used to describe the accumulation of colloidal components (such as the soluble or colloidal fraction of COD) or microorganisms. With a constant flow rate, the pressure difference continues to rise as fouling progresses. The three different phase (acclimatization stage, SRT of 40 days, and infinity SRT) are separated by a chemical wash of the membrane in order to compare the effect of SRT on the fouling evolution. During the acclimatization stage, the TMP varied from 0.085 to 0.5 bar. After the first chemical wash corresponding to the 40-day SRT stage, the TMP evolved from 0.081 to 2.1 bar. During the last stage corresponding to the operation of the pilot without sludge extraction (infinity SRT), a progressive membrane fouling was observed, regardless of the tested volumetric organic load. All curves (Figure 8) showed a two-phase evolution. During the first phase, the pressure rose slowly, and the evolution of the pressure over the time (dΤΜP/dt) was around 6 mb/j (0.69 × 10^−2^ Pa/s), whereas during the second phase, the pressure increased quickly to 28 mb/j (3.2 × 10^−2^ Pa/s). This TMP jump was not observed during phase 1, which lasted only 63 days. It was observed around 0.6 b for phase 2 after 92 days of filtration, whereas the phase 3 TMP jump occurred at 0.4 bar after only 60 days of filtration. The condition of total cell retention did not seem to amplify membrane fouling. This observation can be explained by the reduction in soluble COD, which counterbalanced the high value of suspended solids. Nevertheless, the TMP jump occurred earlier.

## 4. Conclusions

In this study, an experimental treatment of cane industry effluent was conducted in an anaerobic membrane bioreactor, highlighting the relevance of anaerobic membrane bioreactor conditions as an effective and intensive treatment. The AnMBR process can operate at a very high organic loading rate, i.e., 19 kgCOD·m^−3^·d^−1^. More than 96% of organic matter (COD) was removed with a low COD effluent concentration (1500 mg_COD_·L^−1^), decreasing over time, even after more than 350 days of operation. The biomass conversion yield (gVSS_formed_/gCOD_removed_) was very low, confirming the relevance of AnMBR in solving the sludge problem and reducing the cost of sludge disposal. During the operation, biogas conversion yield (LCH_4formed_/gCOD_removed_) was close to its theoretical value, confirming that the main treatment pathway is the biological reaction and not membrane retention. The TMP presents a two-phase evolution whereby the pressure slowly increases in the first phase: 0.69 × 10^−2^ Pa/s. A good overview of biological activity and, therefore, the associated gas production is provided by the epifluorescence measurements taken at a wavelength of 600 nm.

Thus, when the SRT is extended, the performance of the membrane bioreactor increases, and the microorganisms are able to degrade the difficult-to-biodegrade fraction of pollution. Biogas production is also favored with a rapid conversion of the feed substrate. High organic loads, together with the high temperatures favored by the Sahelian context, also contributed to considerable biogas production with favorable acclimatization of the microorganisms responsible for the degradation of organic matter. The ideal temperature for anaerobic digestion with biogas recovery in a membrane bioreactor is around 35 °C. This temperature minimizes the amount of equipment that must be installed, as well as the energy spent on heating the reactors in the Sahelian context.

## Figures and Tables

**Table 1:** Analysis methods.

Parameter	Standard Method
Total solids (TS)	APHA 2540 B
Biochemical oxygen demand (BOD_5_)	APHA 5210 B
Chemical oxygen demand (COD)	APHA 5220—D
Microbial density	NF EN ISO 11348-1
Suspended solids (SS)	AFNOR NFT 90-105
Volatile suspended solids (VSS)	AFNOR NFT 90-029
Absorbance	PBS ISO 3696:1987
VFA (volatile fatty acids)	Biogas Titrator for FOS/TAC, Hach
Ferrous ion (Fe^2+^)	APHA 3500—B
Nitrate ions (NO_3_^−^)	APHA 4500—C
Phosphate ions (PO_4_^3−^)	APHA 4500—B
Ammonium ions (NH_4_^+^)	APHA 4500—C
Magnesium ions (Mg^2+^)	APHA 3120B
Sodium ions (Na^+^)	Flame photometry method
Sulfate ions (SO_4_^2−^)	APHA 4500—G
Chloride ions (Cl^−^)	APHA 4500—G
Calcium ions (Ca^2+^)	APHA 3500—B
Bicarbonate ions (HCO_3_^−^)	APHA 2320—B

**Table 2:** Characteristics of sugarcane industry effluents.

Parameter	Minimum	Maximum	Average ± SD
Number of samples	156		
pH	6.8	7.5	7.1 ± 0.2
T°	21.5	42.3	31.9 ± 4.1
Turbidity (NTU)	1455	1966	1710 ± 94
Conductivity(µs/cm)	10,020	12,960	11,490 ± 688
COD (mg/L)	26,070	37,400	31,735 ± 2709
BOD_5_ (mg/L)	16,900	25,890	21,395 ± 2330
COD/BOD_5_	1.48		
1 COD/N/P	100/0.2/0.01		
SS (mg/L)	7360	8960	8160 ± 302
VSS (mg/L)	5010	6120	5565 ± 205
VSS/SS		68.2%	
TS (mg/L)	23,050	77,820	50,435 ± 8140
Color	Brown	Brown	Brown
PO_4_^3−^ (mg/L)	3.4	19.2	11.3 ± 3.7
SO_4_^2−^ (mg/L)	503	699	601 ± 42
Mg^2+^ (mg/L)	200	299	249 ± 23
Ca^2+^ (mg/L)	712	869	790 ± 39
Fe^2+^ (mg/L)	20.4	35.9	27.1 ± 3.7
Total nitrogen (mg/L)	20.2	103.4	61.8 ± 17.7
NO_3_^−^ (mg/L)	1.2	14.1	7.6 ± 2.4
NH_4_^+^ (mg/L)	2.6	13.7	8.1 ± 2.6
K^+^ (mg/L)	16.6	93.2	54.9 ± 8.8
F^−^ (mg/L)	20.5	68.8	44.6 ± 5.3
HCO_3_^−^ (mg/L)	920	1085	1002 ± 29
Cl^−^ (mg/L)	496	916	706 ± 147
Na^+^ (mg/L)	27.1	186.2	106.6 ± 12.1

**Table 3:** Operating conditions.

Hydraulic Conditions	
Reactor volume	20 L
Hydraulic flow rate	12 L·d^−1^
Hydraulic retention time	40 h
Permeate flux	1.1 LMH
Biological conditions	
Temperature: 35 °C	
OLR: 15 to 22 gCOD/L/d	
Sludge extraction Run I (day 0 to day 62): no extraction Run II (day 62 to day 209): Q_W_ = 0.5 L·d^−1^, SRT = 40 d Run III (day 209 to day 353): no extraction	

**Table 4:** Global trend.

Mass Balance					
	Influent	Reactor	Permeate	Extraction	Biogas
Phase 1	376 gCOD/d	2.7 gVSS/d	103.7 gCOD/d	0	13.6 L_CH4_/d
Phase 2	368 gCOD/d	0.825 gVSS/d	85.4 gCOD/d	7.5 gVSS/d	42.5 L_CH4_/d
Phase 3	385 gCOD/d	0.5 gVSS/d	20.5 gCOD/d	0	109.5 L_CH4_/d

**Table 5:** Overall performance.

Parameter	Unit	Phase I	Phase II	Phase III
Running period length	d	0–63	63–209	209–353
Volumetric organic load (C_v_)	kg_COD_·m^−3^·d^−1^	18.84	18.44	19.28
Effluent concentration *	g_COD_·m^−3^	8325	6304	1490
COD removal rate	kg_COD_·m^−3^·d^−1^	13.61	14.13	18.22
Organic matter removal efficiency	%	75%	85%	96%
Observed sludge production rate	kg_VSS_·m^−3^·d^−1^	0.135	0.416	0.025
Conversion yield	kg_VSS_/kg_COD_	0.01	0.029	0.0013
Methane conversion yield	L_CH4_·g_COD_^−1^	0.05	0.15	0.30

* Average over the last ten phase days.

## Data Availability

Not applicable.
